# Parasite vulnerability to climate change: an evidence-based functional trait approach

**DOI:** 10.1098/rsos.160535

**Published:** 2017-01-11

**Authors:** Carrie A. Cizauskas, Colin J. Carlson, Kevin R. Burgio, Chris F. Clements, Eric R. Dougherty, Nyeema C. Harris, Anna J. Phillips

**Affiliations:** 1 Department of Environmental Science, Policy, and Management, University of California, Berkeley, CA, USA; 2 Department of Ecology and Evolutionary Biology, University of Connecticut, Storrs, CT, USA; 3 Institute of Evolutionary Biology and Environmental Studies, The University of Zurich, Zurich, Switzerland; 4 Department of Ecology and Evolutionary Biology, University of Michigan, Ann Arbor, MI, USA; 5 Department of Invertebrate Zoology, Smithsonian's National Museum of Natural History, Washington, DC, USA

**Keywords:** parasite extinction, host–parasite interactions, climate change, conservation, biodiversity, disease ecology

## Abstract

Despite the number of virulent pathogens that are projected to benefit from global change and to spread in the next century, we suggest that a combination of coextinction risk and climate sensitivity could make parasites at least as extinction prone as any other trophic group. However, the existing interdisciplinary toolbox for identifying species threatened by climate change is inadequate or inappropriate when considering parasites as conservation targets. A functional trait approach can be used to connect parasites' ecological role to their risk of disappearance, but this is complicated by the taxonomic and functional diversity of many parasite clades. Here, we propose biological traits that may render parasite species particularly vulnerable to extinction (including high host specificity, complex life cycles and narrow climatic tolerance), and identify critical gaps in our knowledge of parasite biology and ecology. By doing so, we provide criteria to identify vulnerable parasite species and triage parasite conservation efforts.

## 1. Introduction

Rapidly changing climates are widely recognized as a major contributor to the sixth mass extinction event in Earth's history [1], and the potential impacts on ecosystem form and function are severe and likely to be irreversible [2]. With estimates of up to 54% of free-living species eventually committed to extinction [3], the fate of parasites remains uncertain, despite significant research profiling climate-driven biodiversity loss since the turn of the century. Like most invertebrate species [4], parasites are poorly catalogued in biodiversity risk assessments, which are often biased towards the conservation of more charismatic vertebrates [5].

When parasites are considered in the climate change literature, the majority of studies focus on virulent pathogens that could become dominant in a changing climate, raising human health concerns [6–8]; but the majority of parasites have no direct effect on human health, and the potential negative impacts of climate change on most wildlife parasites are, by and large, empirically untested. Macroparasites, which we focus on here, are already uniquely sensitive to secondary extinctions; theoretical work suggests that parasite vulnerability may be 10 times higher than the baseline extinction rate of their hosts due to the diversity of parasites relative to their hosts and the high potential for secondary and tertiary extinctions [9]. Consequently, the high extinction rate vertebrates face under climate change should be matched by an accompanying mass coextinction, but that phenomenon is poorly documented at best [10].

Moreover, coextinction is only a fraction of the total vulnerability parasites face. Some species go extinct before their hosts or concurrent with host population decline (e.g. *Colpocephalum californici* lice on the California condor, *Gymnogyps californianus* [11]). But as we highlight here, some parasites might experience decline due to the direct pressures of climate change, entirely separate from hosts. Like all multicellular organisms, parasites have an ecological niche that includes climatic constraints to survival and reproduction. Among many examples in the literature, which we highlight throughout the rest of the paper, the geographical boundaries and ecology of ectoparasites can be affected by aridity [12], salt spray [13], elevation and cold [14, 15], while endoparasites can be affected by precipitation, soil type, temperature and other variables [16, 17]. A changing climate alters the availability of parasite niche space, driving a combination of habitat loss and range shifts, and potentially decreasing population growth and reproductive rates, all potentially encouraging primary extinctions.

**Figure 1.**
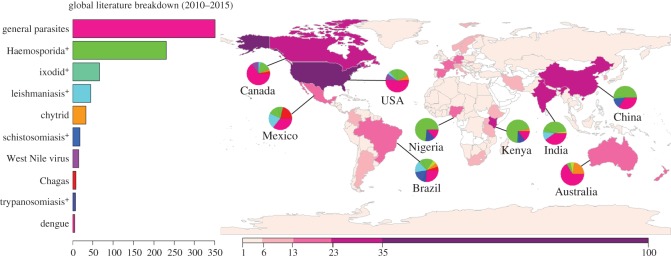
Global distribution of parasite climate change research. Research on parasitic species is disproportionately oriented towards human emerging infectious diseases (EIDs), especially in countries where the majority of parasite research occurs. The figure shows the distribution of 649 studies on parasite ecology (excluding pest management, plant parasites, and reviews, reducing 2200+ studies from Web of Science between 2010 and 2015 featuring the keywords ‘parasite’ and ‘climate change’ down to relevant primary literature) by country and by study system. To illustrate the disciplinary focus on EIDs, major research topics such as haemosporidian blood parasites (primarily malaria) and ixodid ticks and diseases they carry (such as Lyme borreliosis) are separated out from general parasite ecology studies. Global and continental modelling papers were not plotted as they only amplified the focus on major infectious diseases. Combined with a few other diseases such as leishmaniasis, schistosomiasis and trypanosomiasis (and associated vectors and reservoir hosts for each, shown with a ‘+’), the major human EIDs easily match the volume of the entire remainder of climate change literature within parasitology as a discipline. Even in this small subset of the literature, the asymmetry of parasite ecology studies as they relate to climate change is evident.

That loss of parasite biodiversity may have cascading effects in resource–consumer webs, and change the productivity and stability of ecosystems in unpredictable ways [18] that are often overlooked in the literature (figure 1). Some estimate that up to 70% of all animal species are parasites [19], making parasitism the most common consumer strategy on Earth [20, 21], and parasites account for a significant portion of biomass [22] and up to 78% of food web links [23] in any given ecosystem. Their presence and diversity has also been suggested as an indicator of ecosystems with a low degree of human degradation [24]. In addition, evolutionary specializations like host behaviour modification can increase biomass flow between free-living hosts up to 20-fold [25], while adaptations like host castration can drastically limit host population size [26]. Recent research has also highlighted that within-host interactions and competition between parasites can dilute disease risk for hosts in counterintuitive ways [6, 27]. As demonstrated in the *Ribeiroia* trematode–amphibian experimental system [6], parasite and host biodiversity can dilute both disease risk and parasite-induced host mortality at the population level. Just as decreasing diversity in free-living species often increases the dominance of the most abundant species, parasite extinctions could have unpredictable effects on the structure of disease communities, as some pathogens could experience competitive release as rare species go extinct.

The importance of parasites in these ways is well established, and now, as Strona [28] directs in a recent review, we must consider what happens when parasites vanish. We anticipate the parasite biodiversity loss, and associated downstream ecosystem consequences, may be a significant crisis of planning for conservation. While Dougherty *et al.* [29] highlight the methods for conserving known vulnerable parasites, the 300 000 species of helminths alone pose a significant problem of triage [21]. With the exception of three species (*Hematopinus oliveri*, the pygmy hog louse; *Acizzia veski*, Vesk's plant louse; and *Hirudo medicinalis*, the medicinal leech), no other parasite species have been included in the IUCN Red List. Parasites are not unique in this respect [30], as IUCN assessments had only covered 0.3% of invertebrate diversity in 2007, and the same data deficiencies have continued to limit risk assessments over the last decade [31]. By 2015, the IUCN estimates that less than 0.1% of contemporary animal species have already gone extinct, but accounting for poorly documented groups that figure is probably closer to 7%, highlighting the severity of data deficiencies in invertebrate conservation [32]. Identifying traits that most predispose parasites to climate-driven extinction, separate from their hosts, would provide conservation a much needed framework for risk assessment that is robust and applicable across major parasitic groups.

**Figure 2.**
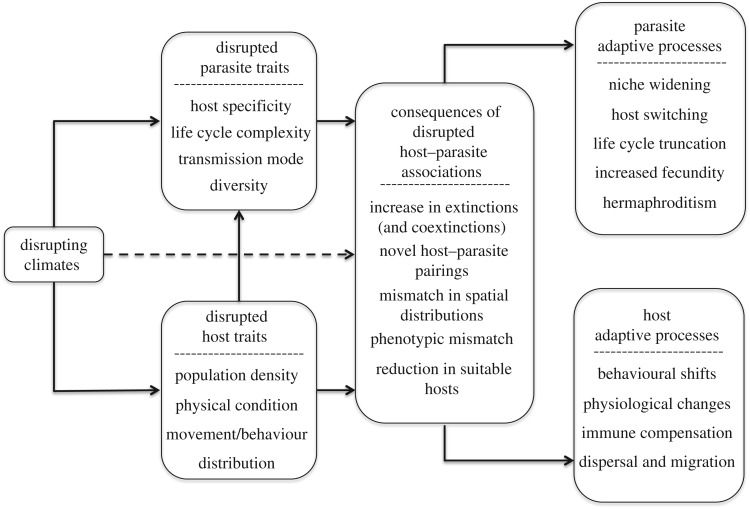
Parasite traits and abiotic and biotic interactions leading to parasite vulnerability under climate change. We list the most important biological traits of parasites that could amplify their vulnerability to extinction under climate change, the most likely changes to host–parasite interactions and various mitigation strategies likely to be used by both parasites and hosts adapting to disrupted climates.

In this review, we highlight the aspects of parasite biology that make this (polyphyletic) category of organisms particularly vulnerable to extinction resulting from climate change, and identify biological traits of parasite species that may act as important predictors of different outcomes under climate change (summarized in figure 2). Those hypotheses are loosely focused on helminth endoparasites, but are also readily applicable in many cases to other parasitic groups (e.g. in discussions of host specificity or free-living stages). Many of our results come from theoretical work (which is often applicable across different forms of parasitism) or generalized patterns in the literature, and has enabled us to identify what we believe are testable and falsifiable hypotheses. As Houlahan *et al.* [33] note in a recent and incredibly valuable review of the role of prediction in ecology, ‘A hallmark of ecological research is that we test coarse hypotheses that have relatively low information content’. With the relationship between climate change and the many aspects of parasite biology we cover here often unexplored for various clades, we focus on these generalized, testable hypotheses, which can be refined and tailored to the incredible diversity of parasitic life on Earth. We also outright acknowledge the massive data deficiencies characterizing many parasite species and clades. Thus, in addition to our framework, we devote the final section of our paper to identifying the major missing links within each discipline that are needed to build an interdisciplinary parasite conservation toolbox.

## 2. Predictors of parasite vulnerability

As is characteristic of most invertebrates, the sizeable fraction of undescribed or unstudied parasite species [21] has, generally, prevented the same sorts of comprehensive vulnerability risk assessments for parasites that have been attempted for a subset of free-living macrobiota (i.e. PREDICTS database [34]). Geographic distributions and population size are the most reliable predictors of climate-induced extinction risk for many IUCN Red Listed free-living species [35], but these data are lacking for most parasite species. To guide the development of conservation efforts that include parasites [29], we outline important biological traits of parasites that affect their success in the face of climate change (summarized in figure 2). We consider four major mechanisms by which climate change will influence the persistence odds of parasites, and for each mechanism, our approach seeks to go beyond identifying basic drivers of parasite vulnerability and instead proposes a series of testable hypotheses to identify parasites that will thrive or suffer under climate change:

1. *Metabolic ecology*. Parasites less buffered from unpredictable ambient temperature fluctuations by poikilothermic hosts will, with some exceptions, be the most vulnerable;
2. *Host body size*. Parasites of larger hosts will be more vulnerable to extinction due to increased coextinction risk and the subsequent loss of a large number of parasite niches and parasite diversity;
3. *Host specificity and host switching*. Host specific parasites face overall higher risks of coextinction, but hidden plasticity enabling host switching may alleviate some of those risks;
4. *Transmission and persistence*. Each independent stage in a parasite life cycle, either in different hosts or free-living stages, can be characterized by its own ecological niche; the overlap among them can become spatially or phenologically disjunct, compounding vulnerability.

It is important to stress the interdependence of these four predictors and the synergistic interactions between them, which may be strongly correlated for many clades. For instance, monogenean parasites have a direct life cycle (low risk) but a strong correlation between host body size and host specificity [36], potentially predisposing them to high extinction risk. Each of those factors individually acts as a driver of vulnerability, and as a suite they may highlight some of the parasites we warn could be most affected by climate change.

### 2.1. Metabolic strategies

At the simplest level of adaptation, endoparasites are affected by climate change through the physical environment within their hosts. In the face of climate change, hosts capable of adapting to new temperatures may escape parasitic infection through ‘thermal refugia’ in which parasites can no longer viably persist. In many cases, this is not an issue of absolute tolerance limits, but instead of steady declines in fitness. Work by Ibelings *et al.* [37] highlights that even highly virulent parasites can have upper thermal bounds above which hosts can escape infection, and while what they call the ‘warmer hence sicker world’ hypothesis is widespread in the literature, climate change can have a far more unpredictable effect on host–parasite dynamics.

The relationship between host and parasite thermal ecology is complicated by trade-offs between host adaptation and immunity, which varies directly with both body size and form of thermal homeostasis (i.e. homeothermy versus poikilothermy). Parasites in homeothermic hosts may benefit from external temperature fluctuations; homeotherms may expend more energy to maintain internal temperature within the optimal part of their performance curves, limiting their immune resource expenditure for anti-parasite defences and reactions [38, 39]. Conversely, parasites of poikilothermic hosts may be more vulnerable to fluctuating temperatures (see electronic supplementary material, figure S1), because poikilothermic internal temperature changes widely with environmental temperature, but coevolutionary processes may already drive a matching degree of eurythermality between hosts and parasites. However, as illustrated by experiments on antimicrobial responses to chytrid fungus (*Batrachochytrium dendrobatidis*), unpredictable temperature changes may compromise host immunity far more severely than long-term warming trends [40], and this would probably ultimately be to the benefit of parasites in poikilothermic hosts. Thus, the higher extinction rate that poikilothermic vertebrates like reptiles and amphibians are generally assumed to face may not be matched by primary extinction risk for parasites, and other traits (like the ones we describe in other sections below) will be more suitable within-clade predictors of parasite vulnerability.

Finally, for parasites with free-living stages, environmental conditions most directly influence parasite survival, and the physiological responses of free-living stages to temperature changes may be a better predictor than any host traits. For example, excessively high temperatures can cause physiological stress in free-living parasites and thus mortality [41], as well as desiccation of eggs and larvae [42]. Alterations to rainfall patterns, and thus water availability, could be detrimental, particularly for those parasites that require inundation to complete life cycles or moisture to facilitate environmental survival and transmission. Pickles *et al.* [43] found that while temperature of the coldest month of the year was the most important variable for determining the distribution of North American white-tailed deer (*Odocoileus virginianus*), the definitive host for the meningeal parasitic worm (*Parelaphostrongylus tenuis*), precipitation in the warmest quarter was the most important variable for determining the distribution of the free-living *P. tenuis* larvae. Consequently, we argue that the first step in identifying vulnerable parasites is to profile endoparasitic fauna with both free-living stages and large-bodied poikilothermic definitive hosts (e.g. crocodilians or elasmobranchs).

### 2.2. Host body size

Larger hosts often harbour a greater richness of endoparasites (as shown by some work in ungulates [44]), as they have more available niches, tend to have longer lifespans and have metabolic traits (e.g. greater overall energetic reserves that support the high cost of maintaining more parasites and that make them less dependent on steady and constant food availability), that together make them a potentially more stable, long-term parasite habitat. Larger host species also tend to have higher energetic requirements matching the high costs of maintaining more parasites [45]. The more stable internal environment of larger hosts also supports higher host specificity [36], a trait already associated with the higher proportional richness due to the asymmetric nature of host–parasite webs (i.e. specialist parasites favour hosts with greater parasite diversity, a finding that is similarly robust in plant–pollinator and other association networks) [46]. Consequently, evidence suggests larger hosts are likely to host a greater overall richness of more specialized endoparasites, a finding confirmed across different groups, e.g. metazoan parasites of carnivores [47] and fish [48]. Though some individual studies on ectoparasites like fleas have sometimes failed to find the same pattern (e.g. [49]), other studies including all parasites of mammals, including microparasites like viruses, found a similar body size–richness scaling pattern [50].

While large vertebrate hosts may hold the majority of parasite diversity, they are also more likely to adapt slowly to climate change [51], and greater body size is conventionally associated with higher extinction risk [52]; larger hosts may thus be more likely to suffer primary extinction and simultaneously lose their parasites [53]. While we recognize that host body size is only a proxy for parasite vulnerability, the fact that larger-bodied wildlife species face a higher risk of extinction [54] recommends such a metric for pragmatic reasons.

### 2.3. Host specificity

In a framework where specialism is the primary driver of coextinctions, a combination of data on host susceptibility to primary extinction and parasites' host specificity may appear the best predictor of parasite extinction risk [55]. However, current parasite ecology literature makes mixed predictions on whether generalist or specialist parasites face higher extinction risks. Generalist parasites are capable of exploiting more than one, often several, host taxa, while specialists have co-adapted closely to their hosts and are dependent on one or two phylogenetically closely related host taxa for their development and survival. At first glance, specificity should drive coextinction, a finding easily shown with theoretical models [45, 56]. A theoretical food web alteration study found that specialist trematodes in a southern Californian marsh food web were very sensitive to secondary extinctions due to the fact that 64% of these parasites depended on a single host species during at least one of those stages. In 18% of the theoretical scenarios in this study, the extinction of a free-living host species led to the secondary extinction of a parasite, with extinctions of the snail host *Cerithidea californica* responsible for the complete extinction of 17 trematode species [57]. Strict host specificity also often means that a smaller number of suitable hosts are available at any stage in the life cycle, and specialists on unstable host populations should face the highest extinction risk [58].

Finally, specialist parasites require an evolutionary host switch to colonize a new host, whereas generalist parasites by definition have already crossed multiple host-species barriers [59]; host switching is time costly, with rapidity dependent on factors such as host group size and host and parasite spatial overlap [60]. Other studies have found that host switching may not be the primary factor determining parasite extinction, and that generalist parasites may be less vulnerable than specialists by resisting extinction in other ways such as evolving more quickly to avoid host immune defences [61].

However, the specificity–coextinction relationship is far more complex on closer observation. The most significant disconnect between specificity and extinction risk can be attributed to the non-independence of host extinctions. Strona *et al.* [62] found that the most specialized fish parasites had a lower coextinction risk than did more generalist fish parasite groups, probably because specialism is favoured as an evolutionary strategy on hosts with less demographic stochasticity. If parasites are associated with several closely related host species (e.g. multiple hosts in a single genus), covariance of extinction drivers between phylogenetic kin can produce comparable extinction outcomes [63]. Finally, specialist helminths are often very well-represented within their host communities, whereas generalists are usually considerably less abundant in an ecosystem (i.e. lower intensity of infection in a single host) [45].

At high enough host extinction levels, generalists may face comparable or higher extinction rates than specialists, as in island ecosystems after the arrival of humans, where generalist ectoparasites went extinct with approximately 70–80 related bird host species [64]. Similarly, a recent study exploring the distribution of single versus multi-host parasites found that threatened ungulate species harboured a higher proportion of specialist parasites than non-threatened ungulate species, perhaps due to less frequent interactions between host species as their abundances decline [65]. However, this relationship between extinction risk and parasite specificity did not manifest in carnivore species, a result that others have emphasized as indicating that parasite losses may be a purely stochastic process [66]. Debate about the interpretation of these findings is ongoing [67].

Any framework for parasite extinction risk based on specialism also has to acknowledge that parasites that can adapt to novel hosts, or expand geographically into regions with stable hosts, will be far less prone to coextinction (e.g. [68]). In some ecosystems and under certain climatic conditions, due to the limited availability of certain hosts or competitive exclusion by other parasites [69], some parasites may appear as functional specialists or ‘faux specialists’ [70]. When faux specialists encounter changing environments in their native range or novel environments when their ranges shift, species with the necessary potential for adaptation undergo a process of host-switching that the ‘Stockholm paradigm’ of parasitology terms *ecological fitting*. That process, according to Malcicka *et al.* [71], relies on a combination of three baseline processes: phenotypic plasticity of genetically coded traits, correlated trait integration which allows rapid multidimensional phenotypic shifts, and phylogenetic conservatism of traits that offer ‘latent potential’. Together these engender an untapped potential for persistence in ‘sloppy fitness space’ after host extinction, or after misalignment of phenology or distribution between hosts and parasites.

The Stockholm paradigm, and theories of ecological fitting, suggest a glimmer hope for some parasites in the face of a changing climate [72] (and casts doubt on the relative predictive power of coextinction models). In the evolutionary long term, climate change could drive diversification; some authors have argued the staggering diversity of parasites worldwide may have been facilitated by climate-driven periods of intense host switching [73], and experimental [74] and historical [75] evidence further supports that these processes have dramatic effects on the persistence of parasites in unlikely environments. Moreover, as some parasites do poorly, their absence may trigger competitive release in others, enabling a once-excluded species to exhibit plasticity and dominate under novel conditions in a given host species [76]. In the works of Hoberg and Brooks, the process of ecological fitting is interpreted as a driving factor not just in the production of a host–parasite coevolutionary geographical matrix, but also in the emergence of human and wildlife infectious diseases. But they similarly acknowledge that ecological fitting is not without constraints; long-term evolutionary recovery may still be matched by short-term decreases in diversity (i.e. many species are still likely to face some threat of extinction). We further note that the unprecedented velocity of current climatic change may make ecological fitting an ineffective silver bullet for mitigating short-term extinction risks in more species than usual, especially considering the limited success in keeping pace with climate change that most free-living species are already projected to have (see fig. 4 and 5 in [77]). In the light of this evidence, coextinction estimates based on binary host–parasite association matrices could be perhaps replaced by more informed predictive approaches that anticipate host switching. Host switching and parasite diversification occur more often within definitive hosts of the same guild than among hosts of different guilds [69], and are less likely if no closely related hosts occur in the parasite's geographical range or host genera are species-poor [78]. Merging geographical and phylogenetic data may offer a better perspective on the extinction risk of different species in groups of conservation interest.

### 2.4. Distributional shifts

Parasites have limited or no independent dispersal capability and rely upon a host for dispersal, and hosts with significant dispersal ability may buffer their parasites against extinction or even facilitate invasions [79]. For example, Choi *et al.* [80] examined 17 species of migratory birds and six species of ticks, and found that these birds, through changing migration patterns, transported at least two tick species (*Haemaphysalis formosensis* and *Haemaphysalis concinna*) that had not been found previously at the migratory stopover sites. However, parasites with complex life cycles may fail in colonization if changes to host distributions shift or migration routes take them to areas with suboptimal or lack of suitable hosts at any stage, or with intolerable environmental conditions for free-living stages.

If too few parasites migrate with their hosts, parasite density in the new habitat may be too low to maintain occupancy [81]; indeed, an increasing body of literature suggests that parasite range shifts may lag behind host range shifts [82], though this phenomenon is not limited to host–parasite interactions [83]. For example, Hopper *et al.* [84] found that a large marine snail (*Kelletia kelletii*) that had expanded its range northward along the coast of California due to climate change was 20% less likely to be parasitized in this new territory, and harboured only 14% of the parasite species found in snails in its historical range. Similarly, in a meta-analysis of 26 host species across several phyla that had experienced range expansions, Torchin *et al.* [85] found that the parasite richness in native populations was at least twice that found in hosts with climate-facilitated range shifts. Unpublished work by Carlson *et al.* further confirms that parasites are likely to face substantial range loss in a changing climate, regardless of the possibility of host switching, to the point that one in 10 species could be directly threatened with primary extinction (data available at pearl.berkeley.edu).

The biogeographic shifts that drive mismatch in host–parasite associations may be matched by changes in the internal host environment, caused by autonomous host–environment interactions. The combined process is complex and potentially unpredictable: by way of example, we present a hypothetical parasite with an intermediate and definitive host and a free living stage, all experiencing a warmer, drier climate (see electronic supplementary material, figure S1). Each species occupies a fundamental niche in what Hutchinson called the ‘biotope’—the complex multidimensional environmental space that is sampled on real landscapes. Geographical range shifts are merely a consequence of species tracking the shifting biotope, but under climate extremes, some parts of species' niches become entirely unavailable. Because parasites exist at the intersection of each host's niche and that of their free living stage, minor loss of area in each can compound to significantly contract the parasite's distribution. But, as hosts move and experience warmer, more-inhospitable environments, their immune resistance may be lowered [38], and while parasites' free-living stages may lose suitable habitat [41, 42], their hosts may support a higher overall level of infection in that smaller range. This may provide a net benefit to parasites, especially those with the evolutionary ability to bypass dependence on intermediate stages. Of parasites with complex multi-stage life cycles, those that are capable of truncating their life cycle complexity suffer the least competitive disadvantage [45].

As one final note, we caution that host–parasite mismatch can occur temporally as well as spatially, an axis of variation not presented in the electronic supplementary material, figure S1. Niche models projected under climate change scenarios forecast phenological mismatches between host and parasites when geographical shifts occur at different rates [86]. Pickles *et al.* [43] projected shifts in the distribution of *Parelaphostrongylus tenuis*, a meningeal nematode of white-tailed deer (*Odocoileus virginianus*) with an intermediate gastropod host. Despite increases of niche breadth for each species, temporal mismatches in habitat suitability arose between life cycle stages, reducing parasite persistence. As this example and other cases of temporal mismatch illustrate [87], parasite vulnerability to extinction is compounded by each additional life stage or required intermediate host, with each needing independent consideration (see [29]). In fact, by simulating the sequential and random extinction of every component of both theoretical and empirical food webs, Lafferty [55] found that increasing the number of life cycle stages by one in a model system negated the added robustness to extinction that would be provided by an additional 12 suitable hosts, highlighting that complex life cycles have a much more readily noticed effect on extinction risk than host specificity.

## 3. Disciplinary synthesis and research directions

After identifying the biological traits that may affect parasite success under climate change and that can guide the prioritization of parasite conservation, we highlight some of the most important unanswered questions for a handful of disciplines regarding what influence climate change may have on parasite vulnerability to extinction. We stress that climate change science and planning for macrobiota conservation is by its nature interdisciplinary and will have valuable contributions to our knowledge about parasite extinction vulnerability, and that the identification of critical data gaps will often need to come from within each field. Telling the difference between a near-extinction parasite and a near-emergent pathogen will probably require answering the following questions.

### 3.1. Population biology: how does population density interact with climate?

Population viability analysis is one of the most important items in the climate change biologist's toolbox [88], but for parasitic species, both within-host density and host density thresholds may exist that predict population persistence [29]. However, the host population dynamics needed to maintain parasite species in the face of environmental change are not well understood. While a number of vector-borne parasites have strong density-driven vector–parasite associations (e.g. [89]), not all parasite population dynamics is as easily predicted by that of their hosts. Wood *et al.* [90] found that marine parasites with long free-living larval stages could disperse more widely and were thus less affected by nearby host density, suggesting that parasites with broader host ranges and/or the ability to disperse farther may have transmission rates that are less tightly coupled to host density. However, to our knowledge, no studies have examined the interaction between spatio-temporal dispersion, host density and transmission rates for parasites on land. It would not be surprising to find similar effects, however, given the fact that these larvae on land (e.g. helminth larvae) travel short distances and survive for only one to two weeks during their free-living development [91].

### 3.2. Evolutionary biology: how does host phylogeny predict parasite extinction vulnerability in the face of climate change?

Most data linking host infection and parasite life history outside of human diseases have been gathered from fish parasites [45], offering only an incomplete basis for extinction risk estimates in most taxa. As vulnerability to extinction will probably be distributed unevenly across clades, we argue that host phylogenies can fill knowledge gaps in the biology of poorly sampled parasites. Poulin *et al.* [92] found some evidence of phylogenetic clustering of host–parasite ‘hotspots’ (host taxa capable of supporting a diverse array of parasite species) and ‘coldspots’ (host taxa relatively depauperate in parasite species richness) for mammalian, bird and fish hosts. These types of non-random patterns of diversity will ultimately determine the situational relevance of phylogenetic data and methods to the prediction of extinction risk. In general, phylogenetically closely related and ecologically similar hosts tend to have similar parasite assemblages [93], and phylogenetic diversity of a host clade is associated with higher parasite species richness within individual hosts [94]. Even for understudied parasite systems, host phylogeny can fill in knowledge gaps to help develop conservation priorities, especially in cases where phylogenetic data act as sufficient proxy for a host's ecological and immunological characteristics.

### 3.3. Community ecology and biogeography: will parasite extinctions be clustered in particular ecosystems?

It remains unclear in the literature whether there are spatial hotspots of parasite biodiversity, but the ability to identify parasite-rich ecosystems could help tailor parasite conservation schemes on a global scale. From the few existing parasite biodiversity studies, there is evidence for such a host–parasite correlation in biodiversity, e.g. areas with higher bird and mammal diversity appear to have a higher diversity of human parasites [55]. However, the complicated relationship between diversity patterns and parasite host specificity confounds these associations. For example, latitudinal gradients alter patterns of parasite diversity [95] and host specificity [96]. Host specificity also may be more variable between ecosystem types than previously thought; for example, the strictness of fish parasite host specificity is different between marine, limnetic and riparian systems [21]. We suggest that identifying parasite biodiversity hotspots—and testing whether these correspond to hotspots of extinction risk—represents a critical contribution to parasite vulnerability research from macroecologists.

### 3.4. Ecological modelling: how do we simultaneously model parasite processes inclusive of abiotic and biotic requirements?

Most of the current modelling has been focused on extrapolating parasite biodiversity loss from corresponding host risk by using theoretical community models; such research has thus far indicated that specialist parasites and hosts at high trophic levels are the least robust to perturbation [55]. These models do not account for the fact that parasite extinction risk is more than a direct cascade from host extinction (an assumption responsible for the low estimate of 3–5% total coextinction risk for helminths in Dobson *et al.* [21]). The use of species distribution modelling has somewhat improved these analyses, but the majority of these studies have been directed only at reservoir and vector species for human diseases, representing a similar bias to the overall parasite–climate interaction literature (shown in figure 1). The simplest of these studies model the potential distribution of parasites independent of their hosts, overestimating parasite distributions and thereby not accounting for the loss of suitable range at each level of host–parasite interactions (see electronic supplementary material, figure S1).

Mechanistic species distribution models (similar to [43]) have the potential to address some of these challenges and are particularly suited to include dispersal limitations for both hosts and pathogens. However, these models are particularly data-intensive, and first require experiments to establish the physiological limits of a parasite species through the entire life cycle [97]. This has already been done with some success for models of *Schistosoma* [98] and *Angiostrongylus* [99], although these models have received criticism for combining data from different species to fill data gaps [100]. Building a seasonal component into distribution models of both host and parasite species is also important given the critical role phenology plays in the persistence of parasite species [101].

Population dynamic and compartmental epidemiological models may also be implemented to help identify vulnerable parasite taxa. For example, a susceptible–infected–resistant (SIR) model for trypanosomiasis that included both vectors and hosts predicted a potentially shifting parasite spatial range [102]. A similar analysis by Ogden *et al.* [103] examined direct interactions between temperature and the rate of disease increase to predict spatial shifts in the Lyme disease ectoparasite vector *Ixodes scapularis*. Mouritsen *et al.* [104] and Studer *et al.* [105] projected the potential collapse of trematode–amphipod dynamics in the North Atlantic and in coastal New Zealand, respectively, using models that linked transmission rate to temperature. Population dynamic models trained on real data have the added benefit of being sensitive to the transmission dynamics of the parasites chosen for the model, as a wide range of potential transmission functions can be added to fit the system's characteristics [106].

## 4. Proposed next steps

We currently lack the necessary information to help us set conservation goals for most parasites: data on species distributions, diversity, phylogeny, host associations and even basic biology remains sparse for many parasites and make comprehensive assessments especially difficult. We propose a functional trait approach to target the most threatened and understudied parasite groups. With enough data, techniques such as machine learning methods can maximize the use of limited phylogenetic and ecological data in the identification of the most vulnerable parasite species in poorly studied, high-diversity clades (similar to those used by Obsomer *et al.* [107]). Of course, these methods, like any other, abide by the garbage-in garbage-out principle [108], whereby analysis of limited data may result in highly biased (though potentially statistically supported) determinations of vulnerability. In a more immediate sense, species-specific parasite vulnerability assessments should be focused where the highest compounded risks are expected.

One final challenge to mainstreaming parasites into conservation involves determining how to implement conservation plans that include endangered parasites. The lack of motivation in preserving parasite diversity is a product of both the extensive challenges inherent in studying parasites and the traditional push toward eradication of parasitic organisms from endangered and commercially valued hosts as well as to reduce human risks (e.g. eradication of the smallpox and rinderpest viruses, and of the guinea worm, *Dracunculus medinensis*). The difficulty associated with eradicating human parasitic diseases may speak to the underlying adaptability and plasticity of the parasitic life cycle, but we caution that the average wildlife parasite lacks the reliable, dense, and widespread host population a human disease like dracunculiasis has. While proposing how to adapt the current paradigms used for free-living fauna conservation to parasite conservation is beyond the scope of this review (see [29]), we stress that parasites are not necessarily covered by the classic focus of conservation biology. Additionally, the ethics that guide which species' extinctions receive priority remains open to discussion. However, without further intervention, a rapidly changing climate will drive the loss of parasite diversity with profound ramifications across scales from individual hosts to ecosystems.

## Supplementary Material

Figure S1. A Hutchinsonian biotope approach to a hypothetical host-parasite association.

## Data accessibility

Data from figure 1 are available at https://figshare.com/s/a4f21531cacee195420e.
