# Supplementary material for: Parasite vulnerability to climate change: an evidence-based functional trait approach
Source: R Soc Open Sci. 2017 Jan 11;4(1):160535. doi: 10.1098/rsos.160535 (PMC5319317; doi:10.1098/rsos.160535)
Supplement: Figure S1. A Hutchinsonian biotope approach to a hypothetical host-parasite association. [file rsos160535supp1.pdf]

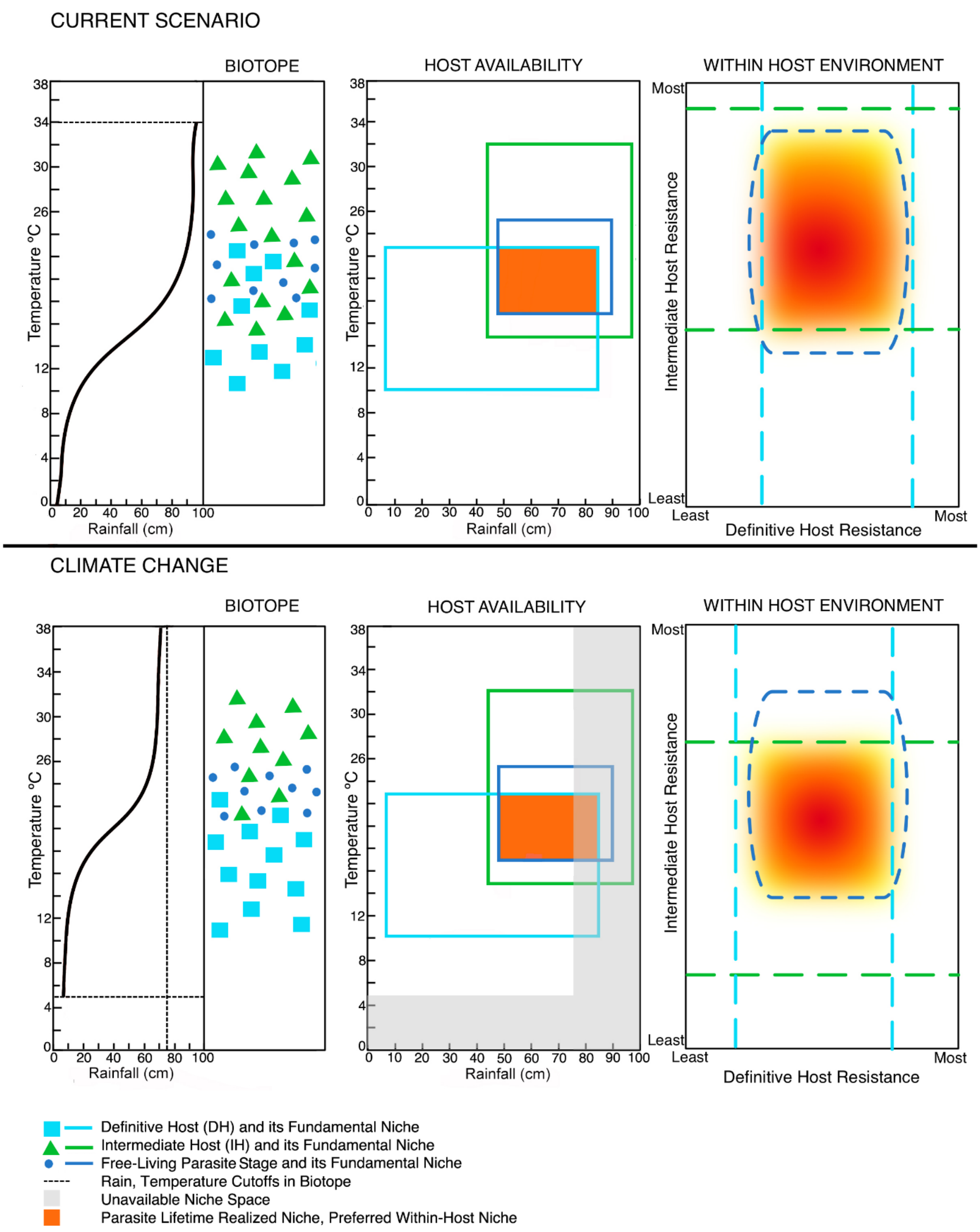

CURRENT SCENARIO
BIOTOPE
HOST AVAILABILITY
WITHIN HOST ENVIRONMENT
Temperature °C
Rainfall (cm)
Intermediate Host Resistance
Definitive Host Resistance
Most
Least
Most
Least
38
34
30
26
12
8
4
0
0
10
20
30
40
50
60
70
80
90
100
CLIMATE CHANGE
BIOTOPE
HOST AVAILABILITY
WITHIN HOST ENVIRONMENT
Temperature °C
Rainfall (cm)
Intermediate Host Resistance
Definitive Host Resistance
Definitive Host (DH) and its Fundamental Niche
Intermediate Host (IH) and its Fundamental Niche
Free-Living Parasite Stage and its Fundamental Niche
Rain, Temperature Cutoffs in Biotope
Unavailable Niche Space
Parasite Lifetime Realized Niche, Preferred Within-Host Niche

Figure S1: *Parasite realized niche size as a function of climate change.* After Figure 1 in Colwell and Rangel [1], we present an example Hutchinson niche-biotope duality [2] for a hypothetical endoparasite species that utilizes a poikilothermic intermediate host (IH) and a homeothermic definitive host (DH), and that experiences a free-living (environmental) stage once in its life cycle. A parasite is dependent not only on external environmental conditions, but also on the spatiotemporal overlap of both of its hosts, and on the internal host milieu that may also shift with climate change. In most populations, the majority of hosts fall within a range of resistant immunotypes, representing a tradeoff between selection from parasitism and the costs of immunity [3]. The poikilothermic IH, due to its reliance in the external environment to maintain its temperature and metabolism, experiences a greater decrease in its immune function than does the endothermic DH. The decreased resistance may act as a boon to the parasite, increasing the intensity and prevalence of infection within the range, despite overall range contractions. This example highlights the need for an approach that accounts for conflicting effects of climate change (positive and negative) at different scales of parasite ecology.

References

[1] Colwell R.K., Rangel T.F. 2009 Hutchinson's duality: the once and future niche. *Proceedings of the National Academy of Sciences* **106**, 19651–19658.

[2] Hutchinson G.E. 1957 Concluding remarks. In *Cold Spring Harbor Symposium on Quantitative Biology*, volume 22, pp. 415–427.

[3] Råberg L., Graham A.L., Read A.F. 2009 Decomposing health: tolerance and resistance to parasites in animals. *Philosophical Transactions of the Royal Society B: Biological Sciences* **364**, 37–49.
